# Global medicine: Is it ethical or morally justifiable for doctors and other healthcare workers to go on strike?

**DOI:** 10.1186/1472-6939-14-S1-S5

**Published:** 2013-12-19

**Authors:** Sylvester C Chima

**Affiliations:** 1 Programme of Bio & Research Ethics and Medical Law, Nelson R Mandela School of Medicine & School of Nursing and Public Health, College of Health Sciences, University of KwaZulu-Natal, Durban, South Africa

**Keywords:** Doctors, Healthcare workers, Strikes, Employees, Employers, Health policy, Law, Ethics, Human Rights

## Abstract

**Background:**

Doctor and healthcare worker (HCW) strikes are a global phenomenon with the potential to negatively impact on the quality of healthcare services and the doctor-patient relationship. Strikes are a legitimate deadlock breaking mechanism employed when labour negotiations have reached an impasse during collective bargaining. Striking doctors usually have a moral dilemma between adherence to the Hippocratic tenets of the medical profession and fiduciary obligation to patients. In such circumstances the ethical principles of respect for autonomy, justice and beneficence all come into conflict, whereby doctors struggle with their role as ordinary employees who are rightfully entitled to a just wage for just work versus their moral obligations to patients and society.

**Discussion:**

It has been argued that to deny any group of workers, including "essential workers" the right to strike is akin to enslavement which is ethically and morally indefensible. While HCW strikes occur globally, the impact appears more severe in developing countries challenged by poorer socio-economic circumstances, embedded infrastructural deficiencies, and lack of viable alternative means of obtaining healthcare. These communities appear to satisfy the criteria for vulnerability and may be deserving of special ethical consideration when doctor and HCW strikes are contemplated.

**Summary:**

The right to strike is considered a fundamental right whose derogation would be inimical to the proper functioning of employer/employee collective bargaining in democratic societies. Motivations for HCW strikes include the natural pressure to fulfil human needs and the paradigm shift in modern medical practice, from self-employment and benevolent paternalism, to managed healthcare and consumer rights. Minimizing the incidence and impact of HCW strikes will require an ethical approach from all stakeholders, and recognition that all parties have an equal moral obligation to serve the best interests of society. Employers should implement legitimate collective bargaining agreements in a timely manner and high-handed actions such as mass-firing of striking HCWs, or unjustifiable disciplinary action by regulators should be avoided. Minimum service level agreements should be implemented to mitigate the impact of HCW strikes on indigent populations. Striking employees including HCWs should also desist from making unrealistic wage demands which could bankrupt governments/employers or hamper provision of other equally important social services to the general population.

## Background

Doctor and HCW strikes have become a global phenomenon with increasing incidence in many countries [1,2] and the potential to impact negatively on the quality of healthcare service delivery and the doctor-patient relationship which is based primarily on the fiduciary duty of trust [3,4]. HCW strikes are not limited to any society, group, or country regardless of their level of socio-economic development. In most democratic societies, strikes are a legitimate part of collective bargaining during labour negotiations [2-4]. Doctor and HCW strikes have been reported in highly developed countries such as USA [2,5-7], UK [8]; New Zealand [9-11], Germany and France [2,12]; middle income countries such as Israel [13,14], India [15], Czech Republic [16], and South Africa [17-19]. Also in less developed countries such as Nigeria [20-22], Malawi [23] and Zambia [24] to name but a few. While HCW strikes occur globally, it appears the impact of strikes are more severely felt in less developed countries because of the poorer socio-economic circumstances and embedded infrastructural deficiencies. Such countries are generally confronted by issues of inadequate manpower, poor wages and working conditions [25], poor organizational ethics [26-28], and lack of viable alternative means of obtaining healthcare for the general population [29], thereby fulfilling the international criteria for vulnerability as defined by UNAIDS and other authorities [29,30].

It has been suggested that doctor and HCW strikes can create a tension between the obligation on doctors and other HCWs to provide adequate care to current patients versus the need to advocate for improved healthcare services for future patients and for society in general [2,31]. There is also a potential conflict between doctors' role in advocating for improved healthcare service for others versus the need to advocate for justifiable wages for self and the fulfilment of basic biological needs like all humans [4,32]. It has been suggested that since strikes are considered a fundamental right or entitlement during collective bargaining and labour negotiations [33]. Therefore to deny any employee the right to strike would be an argument for enslavement of such an employee, because this would simply mean that whatever the circumstances-such an individual must work! A situation deemed to be both ethically and morally indefensible [4]. It is pertinent to observe that there is an on-going paradigm shift in the organization of healthcare services and doctors' employment options with a change in the role of doctors from self-employment, and medical practice based on benevolent paternalism, to consumer rights and managed healthcare [2]. Historically, doctors had the sole responsibility within the doctor-patient relationship, to determine the costs of medical care to their patients, however, current trends show that doctors are increasingly becoming employees of managed healthcare organizations (HCOs) or employees of public health services [2,34-36]. These changes in physicians' practices and methods of payment may impact on patient trust, physician behaviour and decision-making, thereby permanently altering the doctor-patient relationship [3,37]. It has been observed, especially in advanced capitalist societies like the United States, that there is an on-going shift in doctors practice options from self-employment as owners of their own practices [34-36], to doctors becoming employees of HCOs in a managed healthcare environment [2,34,35]. The factors driving this sea change in physicians employment options have been ascribed to "the complex corporate environment coupled with the stress of high malpractice rates, the struggle for reimbursement, administrative duties and the general risks and burden of solo or small group practice" [35,38]. One can therefore anticipate that in the near future there could be more wage negotiations and collective bargaining between doctors as employees and the employing HCOs [35,36]. This will be similar to the practice in systems where medicine is centralized or socialized, and where doctors and HCWs are mostly public service employees [7,10,11,14,16,18,20]. These ongoing changes in the organization of healthcare services and modern medical practice may denote a change in the Hippocratic tenets of the medical profession, creating ethical and moral dilemmas [2,39], which could permanently alter the nature of the relationship between doctors and patients [3,37], and the putative 'contract' between medicine and society [10,40].

## Discussion

### Why do workers go on strike generally?

Strikes are a strategy used by an employee or group of employees in an attempt to force an employer to meet their demands whether economic or otherwise [2,4,41]. Specifically, strikes are used as a deadlock breaking mechanism when employer/employee negotiations have reached an impasse during collective bargaining [4,41,42]. It has been suggested by some authorities that in the absence of the right to strike 'collective bargaining' would amount to nothing more than 'collective begging', in the context that without the threat of a strike or withdrawal of labour, the employee would have no choice than to accept whatever payment or wages proffered by the employer [43]. According to a statement attributed to one physician union leader, doctors not using the right to strike during collective bargaining was similar to a general who says in advance he won't use ground troops in a battle [2]. Therefore, "for a labor movement and collective bargaining to have any force behind it, the right to strike, and ability to do so, is fundamental to ensuring that labor organizing and the bargaining process function and remain credible" [2].

In most countries, the right to strike forms part of statutory law. South Africa is similar since the right to strike is enshrined in the bill of rights and constitution, perhaps as a consequence of the historical struggles for emancipation from apartheid oppression [44]. Strike action as part of collective bargaining is also protected by specific regulations under the South African Labour Relations Act (LRA), when employee grievances cannot be resolved by alternative dispute resolution mechanisms [45]. In more developed countries such as the United States, collective bargaining mechanisms between HCWs such as nurses and their employers have been in existence since 1935 [5,42] and this is supported by federal law under the National Labour Relations Act (NRLA) [46]. Section 7 of this Act, summarizes protected employee activity as follows [42]:

*Employees shall have the right to self-organization, to form, join, or assist labour organizations, to bargain collectively through representatives of their own choosing, and to engage in other concerted activities for the purpose of collective bargaining or other mutual aid and protection*.

Therefore one can agree that striking is a normal and necessary consequence of the organisation of labour markets in capitalist societies [33]. The primary target of a strike action is usually the offending employer, who is expected to suffer damages such as loss of income or negative public relations [2], which would force such an employer to accede to the employee/s demands [4,41]. Collective bargaining has been described as an adversarial process which is designed to win over partial or full control of something that is held by another, especially where wages and improved conditions of service are concerned [3,41,42]. Unfortunately for a strike to be meaningful and effective it must severely affect the employer or a 'third party' who is then expected to bring pressure to bear on the employer to accede to the striking employee/s demands [4,41]. Unfortunately, in healthcare service delivery the affected third parties are usually vulnerable patients and the public who are powerless either because of sickness or lack of alternative means of obtaining healthcare, and who also lack the power to apply the necessary pressure on the employer and employees to break the impasse, due the asymmetric power relationship that exists between patients and the contesting parties, in this case HCWs and their employers [9,20,21,41]. The impact of HCW strikes on the community at large is usually significant [2,17,21,41,47]. It is this negative impact on healthcare service delivery that usually leads to public controversy and opprobrium being heaped on the striking HCWs from both within and outside the medical profession [9,10,18,20,48-51]

### The right to strike as a basic human right

Some authorities have argued that the right to strike can be classified as a fundamental human right because "the right to strike is so important to the functioning of a democratic society that its suppression would be unjustified" [33]. The approach of the International Labour Organisation (ILO) has been to regard this right as a positive right which is subject only to the reasonable restrictions that may be imposed by law [33]. This position is further supported by a legal principle described by Lord Wright in 1942 as follows [52]:

*Where the rights of labour are concerned, the rights of the employers are conditioned by the rights of the men to give or withhold their services. The right of workmen to strike is an essential element in the principle of collective bargaining. It is, in other words an essential element not only of the union's bargaining process itself, it is also a necessary sanction for enforcing agreed rules*.

This sentiment was re-echoed by the constitutional Court of South Africa in the case of *NUMSA *v. *Bader Pop (Pty) Ltd *[53] when the Court opined that:

*The right to strike is of both historical and contemporaneous significance. In the first place, it is of importance for the dignity of workers who in our constitutional order may not be treated as coerced employees. Secondly, it is through industrial action that workers are able to assert bargaining power in industrial relations. The right to strike is an important component of a successful collective bargaining system*.

### Human motivation and the evolution of strikes

**Figure 1:**
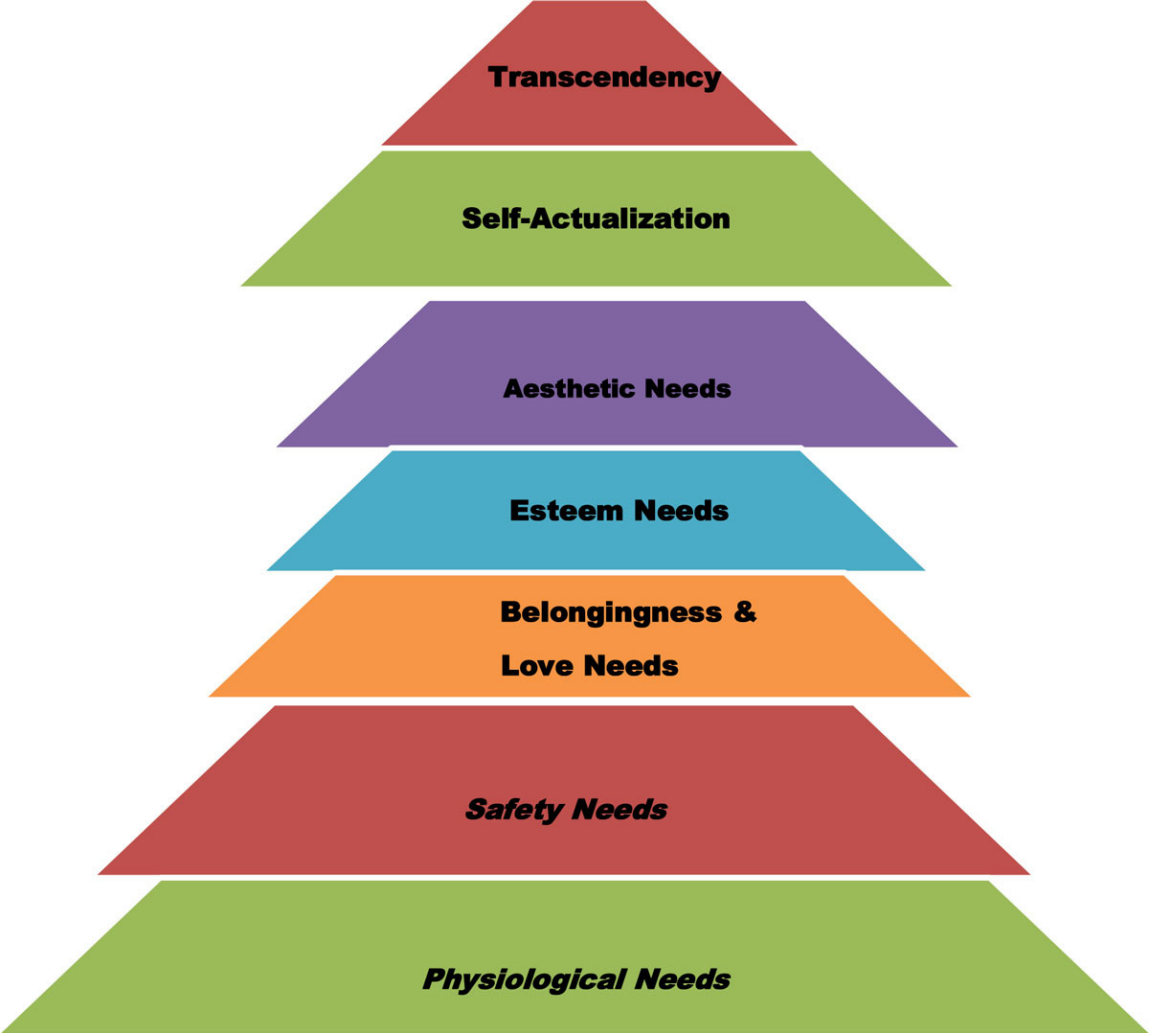
**Maslow's hierarchy of needs for human motivation**. Chima SC, 2013, Adapted from [54].

According to the psychologist Maslow, human beings are generally motivated by the pressure to fulfill certain needs [32]. These needs can be arranged in a hierarchical model ranging from basic physiological needs to self-actualization and transcendence (Figure 1) [54]. Maslow argues further that these human needs may be likened to vitamins in that: (a) One can never be healthy without them, (b) long-term deficiency may cause 'disease' and (c) there are no other substitutes for them. He further suggests that any challenge or possibility of thwarting these basic human needs, or a danger to the defenses, which protect them, or to the conditions upon which they rest, could be considered a threat [32]. It is such threats against the fulfillment of human needs, starting from the basic physiological needs of hunger, shelter etc. that give rise to emergency reactions. One of such emergency reactions, which humans would use defend themselves against a threat to the goal of achieving human needs is a strike action! Based on this analysis one can propose that the higher the level at which a particular community is in terms of satisfaction of basic human needs, the more stable such a community is. Therefore in such communities, the incidence of emergency reactions such as strikes may occur less frequently and their impact is minimized. Conversely, the lower on the level of human development of a particular community, and the lower they are on the ladder towards the fulfillment of the hierarchy of human needs, the more frequent and fierce the struggle to fulfill basic human needs. This may explain why the incidence and impact of strikes is more frequent in developing countries where people are still struggling to achieve basic physiological needs such as food, shelter and healthcare. According to one union leader in South Africa, the primary reason why we go on strike is "our stomach" i.e. to satisfy our hunger or fulfill basic physiological needs.

### Why do doctors and other HCWs resort to strikes?

When doctors and other HCWs embark on strike, three themes appear to dominate the argument presented globally as a reason for their actions. These are generally no different from other causes of doctor disaffection which lead to work attrition or brain drain. According to some doctors leaving KwaZulu-Natal provincial public health services for greener pastures in private practice or overseas, their reasons were; "*working conditions, infrastructure challenges, optimal management, and salaries" *[55]. Thus the reasons given by doctors and HCWs for embarking on strikes may be classified under three themes as follows:

(a) On-going changes in organization of healthcare services beginning from the middle of 20^th ^century to the present [2-5,34-36,38].

(b) Failure by employers to honour collective bargaining agreements for improved wages and conditions of service [3-5,10,18,22-24].

(c) 'Disempowered' doctors and HCWs who feel unable to provide the best possible care for their patients because of inadequate facilities, drugs, and lack of support by employers especially elected government officials [7,12,18,22,26-28]. One can attempt to analyse each of these reasons given for HCWS strikes as follows:

#### The changing face of medical practice and the doctor-patient relationship

The changing face of healthcare delivery and the environment which it is undertaken has brought new challenges to healthcare professionals [32,34-36,38]. Some of these changes include the rise of 'consumerism' in healthcare [37,56-58] and the changing role of the physician from a purely professional role based on beneficent paternalism to that of a service provider and employee in a managed healthcare industry [2,34-36,59]. Starting from the late twentieth century till present, the practice of medicine has changed significantly from its Hippocratic roots. While the requirement of competence endures [60], the doctor-patient relationship has changed, with more knowledgeable and demanding patients. Further, with the legal requirements of informed consent and respect for patient autonomy [57], the patient's welfare is often complex and contested [38]. The obligation of physicians to recommend interventions based on evidence of benefit and harm is challenged by patients who have the expectations of a consumer, in a capitalist and market driven economy [3,34,35,57]. Further, the professional role of the physician as the sole arbiter of patient care has given way to shared decision-making, not only based on the demands of the patient, but on the dictates of the employer, the health care insurance industry, as well as government regulations [34-36]. In the current dispensation doctors have become frustrated and 'disempowered', since their role has been reduced to that of an ordinary worker or employee in many jurisdictions [2]. Even where doctors are involved in private medical practice, their freedom of action is subject to oversight by government officials and regulatory authorities, coupled with willingness or otherwise of the employer or healthcare insurer to pay for the services rendered [35,36]. Because of the emergence of this regulatory framework and the demands of modern society, the doctor has become like any other employee who must occasionally negotiate for increased wages or third party payments to meet personal economic needs. Occasionally such wage negotiations may reach an impasse, demanding resolution by strike action or withdrawal of labor [2,4,11,12]. According to reminiscence by one commentator, forty years ago, before the evolution of managed care, health services were provided as a form of retail transaction. In this scenario, patients went to physician or hospitals of their choice and their employers paid through their group insurance policy. The medical services were based on the cost of each procedure carried out on the patient. The more the services rendered the greater the income for the physician or hospital and therefore the greater their ability to pay their employees or provide better healthcare equipment and services [5,42]. With the advent of managed healthcare, the charges for services are now negotiated at a set rate regardless of the number of procedures at each encounter [42]. This has severely the limited income and compromised the financial resources of doctors and HCOs [2,4,5,34,36]. While the above scenario may not apply to all jurisdictions, other changes in healthcare service industry within the later part of the 20^th ^century have also impacted on the doctor-patient relationship and medical practice generally. For example in the UK, increasing malpractice suits against HCWs and necessary provisions for the clinical negligence scheme for trusts have severely impacted on the amount of money available for patient care services within the national health service (NHS) [61]. Similarly, in less developed countries such as South Africa and Nigeria, recent political changes, poor leadership, and competing demands for limited resources from a large and growing population have impacted on the ability of governments to allocate adequate funds for healthcare service delivery [19-22,27,28].

#### Failure to honor collective bargaining agreements by employers

One of the most frequently cited sources of friction and reason for embarking on strikes is the failure of employers, whether government or private, to adhere to the terms of negotiated wage agreements. For example, in a strike by HCWs in California, USA, nurses and hospital assistants embarked on strike citing their employer for not giving them the 10% salary increase promised, while planning to implement a 25% increase in charges for healthcare services. The workers felt cheated and therefore embarked on a strike action [6]. Similarly, in Philadelphia USA, 1500 striking HCWs claimed longstanding failure by the employer to address issues of staffing levels, patient care, working conditions and also that some HCWs had been working without a valid contract for seven months [7]. In Nigeria, HCW strikes have started following the failure of various state governments to abide with the contents of a memorandum of understanding between the governments and HCWs regarding mechanisms for implementation of a federally negotiated salary scale [22,62]. In South Africa, the public service strikes of 2010 were partly caused by failure of government to implement parts of agreements negotiated with HCWs during previous strikes in 1999 and 2007. The 2007 strike resulted in the introduction of occupational specific dispensation (OSD) salary scales. But partial or shoddy implementation of these agreements as well as refusal by government to agree on a minimum service level agreement was cited as reasons for doctor and HCW strike [18,19,28]. Similarly, strikes in Israel, India, New Zealand, Czech Republic and elsewhere have generally occurred due a quest for improved wages and conditions of service for doctors and other HCWs [8-16]. Therefore it appears that adherence by employers to the terms of wage or conditions of service agreements negotiated through collective bargaining or arbitration may go a long way towards reducing the incidence of HCW strikes.

#### The quest for improved healthcare for delivery for all

It must be recognized that doctors and HCWs are ethically obliged to provide the best possible care for their patients. The Hippocratic Oath to which doctors are required to adhere carries injunction: "*the health of my patient will be my first consideration*" [61]. Therefore in the circumstances where the health of the patient is threatened; for example where there is a failure to provide adequate drugs or proper facilities for patient care. Doctors may feel ethically and morally obliged to intervene on behalf of their patients and this intervention may ultimately result in a strike action or withdrawal of services, in an effort to improve conditions for patient care [7,12,20,22,27,28]. One can argue that the resulting improvement in overall quality of healthcare services when negotiated changes are implemented mitigates any immediate harm of strike actions [2]. Therefore indirectly, strike actions by HCWs may ultimately result in better healthcare for patients and the public in general.

### Doctors and other HCWs as "essential workers"

Despite the fundamental importance of the right to strike in collective bargaining and industrial relations, it has been recognised that derogations or restrictions to this right may be necessary to avoid abuse or usage of this right contrary to the needs of the community [33]. The concept of 'essential service' expresses the idea that certain activities are of such fundamental importance to the community, that their disruption may have particularly harmful consequences to the health, safety or welfare of members of the public [51]. Therefore one of the mechanisms by which governments or elected officials have used to manage the impact of strikes on certain professional groups has been to designate such groups as *"essential workers"*. These employee groups are then statutorily prohibited from striking. In other words they are not allowed to withdraw their labour, regardless of the circumstances. The international labour organization (ILO) has provided a strict list of such "essential services", including *the hospital sector, electricity services, water supply services, the telephone service, the police and the armed forces, the fire-fighting services, public or private prison services, the provision of food to pupils of school age and the cleaning of schools and air traffic control*. However, the ILO list is not exhaustive and a state can add other services to its national legislation if it these are deemed essential to its particular circumstances [33]. Further, the ILO has also used the same criteria to conclude that some jobs are "not essential services". These include banking, agricultural activities, teaching, the petroleum industry, mining, general transport etc. [33]. Based on the above criteria, doctors and HCWs are generally classified as essential workers because it is argued that they have a responsibility that is considered different from other professional groups in society. However it has been suggested that to argue that any particular type of worker should not be allowed to strike is an argument for enslaving such a worker [4,10,51]. Striking means withholding one's labour or professional skills until a particular set of conditions have been met. In a modern society, divided between employers and employees, the only bargaining tool available to an employee or labourer through collective bargaining may be the threat of withholding his or her labour, skills, or services. Therefore denying anybody the right to strike under any circumstances is like saying to that person- no matter the circumstances, you must work! Such employment would be a form of slavery because it denies that individual human dignity and strips them of their freedom of choice by removing the fundamental conditions of liberty and autonomy. This leaves such an employee or worker in a condition akin to slavery and slavery by whatever means has been judged to be ethically indefensible [2,4,10,12,41,51]. One can also argue that denial of such striking rights may also be considered unfair discrimination and therefore morally unjustifiable.

### Philosophical and moral arguments for and against strikes

Some philosophers have described moral obligations or duties, which ought to guide ethical behavior, such as the duty of fidelity or the obligation to keep promises, and beneficence - the obligation to do 'good' [10]. However, it has been suggested that some other equally compelling moral duties or ethical obligations may conflict with the above duties, such as the right to justice. Justice is the right to fair treatment in light of what is owed a person [63]. For example, it may be argued that *everybody is equally entitled to a just wage for just work*. The philosopher Immanuel Kant based his moral theory on a categorical imperative which encourages moral agents to act, based on a principle, which they would deem to become a universal law [64]. One can argue that the decision by any HCW to go on strike may not be universalisable. However, looking at this decision from the principle of respect for autonomy, or freedom of choice, one can conclude that individual autonomy is a sentiment which is desirable for all human beings. Accordingly, every worker should be free to choose whether to work or not, based on a whether any specific set of conditions of their own choosing have been met. Kant argues further that moral agents or individuals should be treated, "whether in your own person or in that of any other, never solely as a means, but always as an end" [64]. This idea that individuals should be treated as ends in themselves has influenced political philosophy for centuries, and stresses the libertarian ideology that people should not have their individual freedoms curtailed either for others or for the good of society in general [10,64]. From this axiomatic considerations, one can conclude that it would be unethical for people to be used as slaves or be forced to work for inadequate wages or under slave-like conditions [4,10,12,51]. The issue of HCW strikes can also be analyzed from utilitarian principles as formulated by one of its major disciples JS Mills as follows [65]:

*The creed which accepts as the foundation of morals, utility, or the greatest happiness principle, holds that actions are right in proportion as they tend to promote happiness, wrong as they tend to produce the reverse of happiness*.

One can argue based on utilitarian principles that the short term suffering induced by doctor and HCW strikes can be mitigated by the long-term benefits such as improvement of healthcare services for the greatest number of people over time [2]. Even if the immediate gains are improved wages and conditions of employment for HCWs alone, in the long-term these will translate into better healthcare service delivery to the local community and society-at-large. Similarly a rights based approach to the issue of strikes, would suggest that even though the goal of bringing about the better healthcare for individual patients or the public at large is a major ethical duty. There is an equally compelling moral duty to protect and enhance individual rights. Protection of individual rights in employment helps to ensure that no group of citizens, are unfairly discriminated against in the quest for equal rights for all in a democratic society.

### The impact of doctor and HCW strikes on healthcare service delivery

#### Impact of doctor and HCW strikes on patients

Contrary to popular belief, withdrawal of services or strike actions by doctors and HCWs will not automatically lead to an increased number of patient deaths or total failure of healthcare service delivery [2,17,41]. In a review of the impact of HCW strikes in different parts of the world, it has been reported that strikes by HCWs may not significantly affect the health of patients, especially where emergency services or alternative service delivery channels such as fee-for-service private care are readily available [2,14,17,41]. More specifically studies from a doctor's strike in Israel and San Francisco USA, showed that while there was increase in the number of patients presenting for emergency care, diagnosis and treatment of specific emergency conditions such as acute appendicitis were not severely impacted by HCW strikes [41]. A more recent study on the impact of strikes on hospital care in a South African hospital showed that there were less hospital admissions during a 20-day strike period when compared to 20-day period non-strike period [17]. Further, the gross number of deaths was reduced, although a statistical projection based on the reduced number of hospital admissions showed that there may have been more deaths statistically [17]. The authors concluded that the strike was associated with reduction in quality of healthcare service delivery [17]. Another study conducted in Sweden during a general strike seemed to show a decrease in mortality rates because of the reduced number of elective surgeries [41]. Other studies on strike impact on mental health services in England, Canada and the USA showed that admission rates for people with mental disorders decreased significantly [41]. A consumer satisfaction survey among group home residents 12 months before and 12 months after a strike by HCWs showed no difference satisfaction level with the community mental health services [41]. Other studies, which investigated the impact of doctors, strike on different socio-economic strata of Israeli society, showed that patients from lower socio-economic groups coped less effectively with strikes and complained of a higher impact on their health as opposed to patients from higher socio-economic strata, perhaps because of affordability of alternative healthcare services. It was further reported that patients from the lower socio-economic classes were less likely to condemn either party in the strike action [14,41]. Perhaps observed low impacts of HCW strikes on service delivery in developed countries could be related to the ready availability of alternative channels for obtaining healthcare such fee-for-service private care and emergency services. Contrary to the low impact of HCW strikes in developed countries, anecdotal evidence from newspaper reports and research seem to suggest that strikes by HCW in developing countries are associated with more patient deaths and have a more severe impact on the general population [17,20,21,23,47]. This is not unexpected considering that these communities may be considered vulnerable populations groups in accordance with UNAIDS criteria for vulnerability [30,31]. This evidence buttresses the need for implementation of minimum service agreements in less developed countries to mitigate the impact of doctor and HCW strikes on the local population, a measure which has been advocated by some local doctors in South Africa [18,19,28,49-51].

#### Impact of strikes on doctors and HCWs

It would appear that strikes may have a disproportionate deleterious impact on doctors and other HCWs when compared to patients. Striking HCWs frequently face a loss of income, job insecurity, and emotional distress, plus long hours of work for those who choose not to participate in the strike action. Further, there could be derangement of working relationships as well as loss of established leadership [11,41]. Whether or not their demands are eventually met, doctors who have been involved in strikes usually end up disillusioned and demotivated and many end-up emigrating overseas or relocating within the country thereby leading to either internal or external brain drain. For example, striking doctors in Timaru, New Zealand reported an "overwhelming feeling of complete lack of confidence and trust in the hospital management team" [11,16,25,55,66]. The impact of such movements could be as severe as occurred in Malta, where the Maltese medical school lost its GMC accreditation due to a prolonged doctor's strike [9]. It could also lead to a situation where close to 25% of a national doctors threatened to quit their jobs and leave the country unless they received wage increases, as reported recently from the Czech Republic [16]. The brain drain which occurred in Malta, New Zealand and Israel following doctors strikes led to major disruptions in healthcare service delivery in the centers and regions affected [9,14].

### Minimizing the incidence and impact of doctor and HCW strikes

One of the most common causes of brain drain from Africa and other developing countries could be traced to the disillusionment engendered by frequent doctor and HCWs strikes leading to worker attrition and perpetuating the cycle of healthcare manpower shortage in these countries [25,28,55,66]. According to one commentator, "healthcare worker motivation is one area in urgent need of attention. A more proactive and progressive policy of motivating providers of health care would ensure we reduce our losses of skilled manpower to other countries. The situation where many Nigerians are left without access to public health services for months on end in some instances as a result of strikes by demotivated health workers is most distasteful, and does not help our already depressing vital statistics" [66]. Further, a report by the working group on human resources for health in Africa has indicated that interventions to stem the migration of health professionals is probably the single most important measure that needs to be undertaken to maintain healthcare workforce in African countries. According this report, "a key action is a significant upward revision of the total compensation package to a level that reflects the value placed on the work they do, is likely to discourage staff from wanting to leave public sector services" [25]. Finally it has been observed that power dynamics existing between employers and employees may provide an impetus to strikes, "an inflexible powerful employer who is unwilling to negotiate on issues considered important by employees, and is more likely to experience job action by employees" [31]. Therefore actions such as recent mass firing of striking doctors by the Lagos state government in Nigeria [62] may not be the best way to resolve employer/employee disputes because such actions will only lead to concomitant court or legal actions by strikers [67], thereby prolonging strikes, resentment, attrition and migration of HCWs from the public services, and defeating the stated purpose of providing effective healthcare services to the local population [55,62,66]. Also, arbitrary punitive actions by regulatory authorities, including the threat of disciplinary action against striking HCWs, such as those proposed the HPCSA [48-50], will only lead to more resentment by doctors and counterproductive results [55]. Therefore employers, employees and regulatory authorities must be ethical in their approach to resolving labour disputes by doctors and HCWs, especially in an environment already plagued by poor quality of healthcare service delivery, poor health outcomes and low confidence in the healthcare infrastructure and public services [19,20,27,55,68].

## Summary

This analysis shows that the right to strike is so important to the functioning of modern democratic societies that its suppression would be unjustified. The right to strike is now accepted as an indispensable component of collective bargaining and perhaps a fundamental human right. However mminimizing the impact of doctor and HCW strikes will require improved organizational ethics and the recognition by both employees and employers, especially elected officials that they are equally morally obligated to serve the interest of society. In other words they are two sides of the same coin. For the incidence of strikes to decrease both employers and employees must be ethical in their approach to resolving labour disputes. For example, legitimate collective bargaining agreements must be respected and honored in a timely manner. Similarly, employees including doctors and other workers must resist the impulse to make economic demands which are beyond the capacity of the employer or which could hamper the provision of other social services, such as education and public utilities. Furthermore when HCWs embark on a strike action, they must endeavor to provide a certain level of minimum service, e.g. continue providing emergency medical services, thereby minimizing the impact of strikes on the general public. In this regard, it is imperative that agreements such as the minimum service level agreements which are being advocated by doctors unions as a means of assuring minimum coverage during strikes should be speedily agreed upon. Governments as employers should also resist the urge to arbitrarily designate certain groups as "essential services", outside of established international law, simply in order to deny such employee groups the right to strike. Arbitrary actions such as mass firing of striking doctors or threats of unjustifiable disciplinary action by regulatory authorities, will not encourage speedy resolution of HCWs, and may lead to undesirable consequences such as brain drain. If some workers or employees are considered 'essential', then society should endeavor to treat such employees as such, by devising mechanisms to pay appropriate wages which justify such 'essentiality'. It may be useful to appoint an independent mediator or administrative body to advice on special salary packages and conditions of service for essential workers rather than grouping every worker together under the rubric of public service employees. Finally, it has been observed following strikes by HCWs in some jurisdictions, that while the public is generally supportive of HCW strikes which are designed to improve the quality of healthcare service delivery for all, society is generally unsupportive of strikes where the sole purpose is the increment of wages and improved conditions for HCWs alone.

## List of abbreviations used

HCW: healthcare workers; HMO: Health Maintenance Organization; HCO-health care organization; HPCSA: Health Professions Council of South Africa; GMC: General Medical Council, ILO: International Labour Organization; JLI: Joint Learning Initiative; LRA: Labour Relations Act; NLRA: National Labor Relations Act (USA); NHS: National Health Service, UK; NUMSA: National Union of Mineworkers, South Africa; UNAIDS: United Nations Agency on Acquired Immunodeficiency Syndrome; OSD: occupational specific dispensation; SAPA: South African Press Agency; UK: United Kingdom; USA: United States of America; SACSIS-The South African Civil Society Information Service; UKZN: University of KwaZulu-Natal.

## Competing interests

The author declares that they have no competing interests.

## Authors' contributions

Author conducted all the research and wrote the manuscript.

## Declarations

Publication of this supplement has been funded by the College of Health Sciences and the Research Office at the University of Kwazulu-Natal.

This article has been published as part of *BMC Medical Ethics *Volume 14 Supplement 1, 2013: Selected papers from the 3rd Ethics, Human Rights and Medical Law Conference (3rd EHMRL). The full contents of the supplement are available online at http://www.biomedcentral.com/bmcmedethics/supplements/14/S1.
